
JOURNAL INFORMATION
==============================
NLM Title Abbreviation: Wirtschaftsdienst
Journal ID: Wirtschaftsdienst
NLM Journal ID: 101086612

Wirtschaftsdienst (Hamburg, Germany : 1949)

ISSN: 0043-6275

ARTICLE INFORMATION
==============================
PMCID: PMC8383012
PMID: 34456389
DOI: 10.1007/s10273-021-2988-0
Article ID: 2988
Article version: 1
Subjects: Ökonomische Trends

Kombination von Kurzarbeit und Qualifizierung — ein gutes Konzept mit mäßigem Erfolg

Combination of short-time work and qualification — a good concept with moderate success

Pusch Toralf Toralf-Pusch@BOECKLER.DE
1
Seifert Hartmut 2
1 grid.473628.c 0000 0001 0945 594X Referat Arbeitsmarktanalyse, WSI in der Hans-Böckler-Stiftung, Hans-Böckler-Straße 39, 40476 Düsseldorf, Deutschland
2 Düsseldorf, Deutschland
Electronic publication date: 2021 Aug 24
Print publication date: 2021
Volume: 101
Issue: 8
First page: 660
Last page: 662
Copyright: © Der/die Autor:in(nen) 2021
License: Open Access: Dieser Artikel wird unter der Creative Commons Namensnennung 4.0 International Lizenz veröffentlicht (https://creativecommons.org/licenses/by/4.0/deed.de).
Open Access wird durch die ZBW — Leibniz-Informationszentrum Wirtschaft gefördert.
License URL: https://creativecommons.org/licenses/by/4.0/

issue-copyright-statement: © The Author(s) 2021

==============================

Kurzarbeitergeld (KUG) bewährt sich in der aktuellen Krise, bedrohte Beschäftigungsverhältnisse zu sichern und Arbeitslosigkeit zu verhindern (Herzog-Stein et al., 2021). Als weniger erfolgreich ist dagegen der Einsatz von KUG in wachstums- bzw. strukturpolitischer Hinsicht zu bewerten. Nur zurückhaltend wird die freigewordene Zeit für berufliche Qualifizierung genutzt; die günstige Gelegenheit, die Qualifikationsstruktur der Beschäftigten an die durch den digitalen, ökologischen und demografischen Strukturwandel ausgelösten Bedarfe anzupassen, wird verpasst. Dabei besteht reichlicher Bedarf, den gesamtwirtschaftlichen Humankapitalstock aufzubessern, wie die OECD (2021) erst kürzlich offengelegt hat.

Günstig ist die Gelegenheit deshalb, weil die Nutzung der durch Kurzarbeit freiwerdenden Zeit für Weiterbildung die Möglichkeit bietet, zwei für Betriebe und Beschäftigte bedeutsame Engpässe, nämlich Kosten und Zeit, zu mindern oder gar zu beheben (Bilger und Käpplinger, 2017). Bei Kurzarbeit verliert die in konjunkturellen Hochphasen mit ausgelasteten Arbeitskapazitäten und Überstundenarbeit knappe Ressource Zeit ihre limitierende Wirkung. Betrieben fällt es leichter, Weiterbildungszeiten mit den für Produktion und Dienstleistung erforderlichen Zeiten zu synchronisieren. Beschäftigte lassen eine größere Weiterbildungsbereitschaft erwarten, wenn die Maßnahmen während der Arbeitszeit (Osiander und Stephan, 2018) stattfinden.

Ebenso kann KUG den Engpass Kosten entschärfen. Für die ausgefallene und stattdessen für Weiterbildung zur Verfügung stehende Zeit wird Kurzarbeitergeld gezahlt. Den Betrieben entfällt ein großer Teil der indirekten Kosten der Weiterbildung, zumal auch die Sozialversicherungsbeiträge erstattet werden. Zusätzlich bieten arbeitsmarktpolitische Programme (Qualifizierungschancengesetz und Arbeit-von-morgen-Gesetz) die Möglichkeit, auch die direkten Kosten (Lehrgangsgebühren usw.) zu senken. Die Kostenübernahme steigt mit abnehmender Betriebsgröße. Für Kleinstbetriebe fallen nahezu keine Kosten der Weiterbildung an.[1]

Auch Beschäftigte können von Weiterbildung während der Kurzarbeit profitieren. Da eine dauerhafte Weiterbeschäftigung nach der Kurzarbeit nicht gesichert ist, können sie mit gezielter Weiterbildung versuchen, ihre Chancen auf dem externen Arbeitsmarkt zu verbessern. Eine erhöhte Mobilitätsfähigkeit und -bereitschaft kann ebenfalls für Betriebe vorteilhaft sein, wenn sie zu selbstinduziertem Arbeitsplatzwechsel führt, sodass bei notwendigem Personalabbau keine ansonsten anfallenden Entlassungskosten (Abfindungen, Sozialplan) entstehen. Umgekehrt könnten Betriebe zurückhaltend reagieren, weil sie das Risiko fürchten, dass qualifizierte Arbeitskräfte abwandern (Crimmann und Wießner, 2009). Weiterbildung kann aber auch die Verbleibchancen von Beschäftigten steigern, da sie durch ihre Teilnahme berufliche Anpassungsbereitschaft und -fähigkeit an sich ändernde Produkte und Produktionsprozesse signalisieren.

Die insgesamt günstigen Zeit- und Kostenbedingungen sprechen für antizyklische Weiterbildungsaktivitäten, für das Nachholen von in konjunkturellen Hochphasen aufgeschobenen oder das Vorziehen von zukünftigen Qualifizierungsbedarfen.

## Bescheidene Teilnahme

Enttäuschend fallen jedoch die Befunde der auf Basis der Erwerbspersonenbefragung der Hans-Böckler-Stiftung durchgeführten Analyse aus (Emmler, 2021).

1 Betriebe mit weniger als zehn Beschäftigten erhalten 100 % der Kosten erstattet, Betriebe mit bis zu 250 Beschäftigten 50 % (Bundesgesetzblatt, 2018).

Kurzarbeiter:innen[2] zum Befragungszeitpunkt November 2020 nahmen seit Beginn der Pandemie seltener (18,9 %) an betrieblicher Weiterbildung teil als Beschäftigte ohne Kurzarbeit (28,8 %).[3] Generell scheint die Arbeitszeit die Weiterbildungsintensität negativ zu beeinflussen. Bei sozialversicherungspflichtig Beschäftigten mit Arbeitszeitverkürzungen von über 50 % (März-Oktober 2020, vermutlich überwiegend durch Kurzarbeit bedingt) sinkt die Teilnahmequote auf lediglich 8,7 %, deutlich weniger als bei Beschäftigten mit weitgehend unveränderten Arbeitszeiten (30,2 %).

Weiterbildung im Zusammenhang mit Kurzarbeit durchbricht nicht tradierte Strukturmuster (Statistisches Bundesamt, 2017; Schönfeld und Behringer, 2017), sondern repliziert sie: Die Teilnahme der Kurzarbeiter:innen (ohne Abschluss: 4 %, mit Berufsabschluss: 14 %, mit Hochschulabschluss: 35 %) steigt mit dem Qualifikationsgrad der Beschäftigten, ähnlich wie bei den sozialversicherungspflichtig Beschäftigten ohne Kurzarbeit im November 2020 (ohne Abschluss: 19 %, mit Berufsabschluss: 25 %, mit Hochschulabschluss: 36 %), ebenso mit der Betriebsgröße, woran auch die umgekehrt zur Betriebsgröße gestaffelten öffentlichen Fördermöglichkeiten (Qualifizierungschancengesetz) im Grundsatz nichts geändert haben. Höher ist die Weiterbildungsbeteiligung zudem in Betrieben mit betrieblicher Interessenvertretung als in solchen ohne. Ferner zeigen sich starke Branchenunterschiede, unterdurchschnittliche Werte vor allem in den stark von KUG betroffenen Bereichen wie dem Gastgewerbe mit 12,5 % (vgl. Abbildung 1). Wenn in diesem Bereich die Weiterbildungsquote der Beschäftigten mit Kurzarbeit höher ist als bei denen ohne Kurzarbeit, könnte das mit Abwanderungen aus diesen Tätigkeiten und der Vorbereitung auf neue Anforderungen zu tun haben.

Grosso modo bestätigt werden diese Befunde für den Zeitraum der Corona-Pandemie durch eine Regressionsanalyse. Beschäftigte mit einer längeren Dauer der Kurzarbeit nehmen signifikant weniger an Weiterbildung teil (Pusch und Seifert, 2021). Auch für den Grad der Qualifikation der Beschäftigten (positiv für Weiterbildungsteilnahme) und bestimmte Branchen (Kommunikation, Finanzen, Gesundheits- und Sozialwesen) gibt es signifikant positive Zusammenhänge mit der Weiterbildungsaktivität. Im Gesundheitswesen dürfte die hohe Weiterbildungsquote mit den Anforderungen der Pandemie zu tun haben. Im Gegensatz zur Tarifbindung gibt es für das

Abbildung 1

**Weiterbildungsteilnahme Beschäftigte mit und ohne Kurzarbeit, nach ausgewählten Branchen**

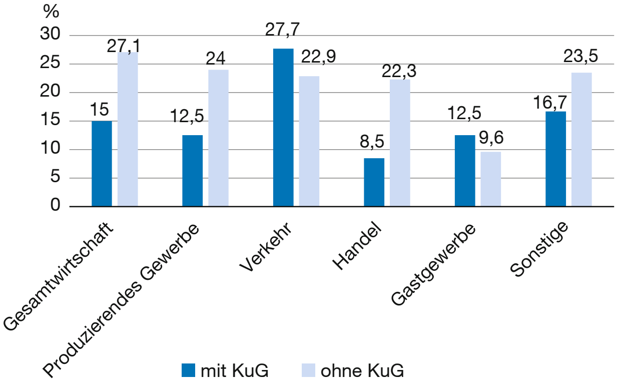

Quelle: HBS-Erwerbspersonenbefragung, eigene Berechnungen.

Vorhandensein eines Betriebs- oder Personalrats einen hoch signifikanten Zusammenhang mit der Weiterbildungsaktivität. Insgesamt lässt sich nicht sagen, ob die zurückhaltenden Weiterbildungsaktivitäten eher auf eine mangelnde Bereitschaft der Beschäftigten oder der Betriebe zurückzuführen sind. Wie Erfahrungen zeigen, machen Betriebsräte in Krisensituationen Weiterbildung zu einem Thema (Berger, 2015) und stoßen entsprechende Initiativen an, um bedrohte Arbeitsverhältnisse zu sichern.

## Gründe für die Zurückhaltung bei der Weiterbildung

Die Weiterbildungsintensität hängt von generellen und von kurzarbeitsspezifischen Faktoren ab. Generell bereiten die coronabedingten Kontaktbeschränkungen Schwierigkeiten, Weiterbildung durchzuführen (Christ und Koscheck, 2021; Bellmann et al., 2020). Zudem ist die Mehrheit der Betriebe nicht über Angebote öffentlicher Förderung informiert, oder aber Beschäftigte (30 %) zeigen kein Interesse an den angebotenen Maßnahmen (Kruppe et al., 2021).[4] Von der Krise betroffene kleinere und mittlere Unternehmen haben ihre Weiterbildungsaktivitäten eingeschränkt oder gar eingestellt, weil das Weiterbildungsangebot beeinträchtigt ist, eigene Planungskapazitäten fehlen, sie die Zukunftschancen als unsicher einschätzen oder der kurzfristigen Existenzsicherung Vorrang einräumen (Leifels, 2021). Zwar haben Weiterbildung betreibende Betriebe alternative Lernformen wie E-Learning ausgebaut, sie sind aber noch längst nicht flächendeckend im Einsatz (Seyda und Placke, 2020). Au-

2 Gezählt wird die Teilnahme an einer Weiterbildungsmaßnahme in der Zeit von Beginn der Corona-Pandemie bis Anfang November 2020.

3 Für Juni 2020 beziffert eine andere Untersuchung den Anteil der Kurzarbeiter:innen mit Beteiligung an Weiterbildung auf 5 %, ohne Vergleichswerte für Nicht-Kurzarbeiter:innen (Kruppe und Osiander, 2020).

4 Es wird allerdings nicht zwischen Beschäftigten mit und ohne Kurzarbeit unterschieden.

ßerdem ist fraglich, inwieweit E-Learning für weniger bildungserfahrene Personen geeignet ist.

Bei Kurzarbeit kommt erschwerend hinzu, dass Qualifizierungsmaßnahmen einen planerischen und organisatorischen Vorlauf erfordern (Deeke, 2009), der zumindest in der Anfangsphase der coronabedingten Kurzarbeit im Frühjahr 2020 nicht gegeben war. Mit andauernder Kurzarbeit verliert dieses Hindernis allerdings an Bedeutung. Problematisch bei der Organisation von Weiterbildung ist, dass die Dauer der Kurzarbeit in vielen Fällen nicht absehbar ist, Maßnahmen nicht in die durch Kurzarbeit veränderten Arbeitspläne passen oder auch nicht als prioritär angesehen werden (Bellmann et al., 2020). Außerdem entfällt bei Kurzarbeit mit einer stark reduzierten Arbeitszeit weitgehend der Lernort Betrieb. Gerade aber das Lernen am Arbeitsplatz hat eine hohe Bedeutung in weiterbildenden Unternehmen.

## Fazit

Das *window of opportunity*, Weiterbildungsdefizite während der Kurzarbeit nachzuholen, wird nur bescheiden genutzt. Offensichtlich fällt es Betrieben und Weiterbildungsanbietern schwer, geeignete Maßnahmen zu organisieren. Auch verfügen Betriebe und Kurzarbeitende häufig nicht über die erforderlichen Informationen über Qualifizierungsmöglichkeiten, -inhalte, -träger sowie öffentliche Fördermöglichkeiten. Gezielte Beratung ist deshalb das Gebot der Stunde. Offensichtlich reicht die mit dem Qualifizierungschancengesetz ausgebaute Beratung nicht aus, modularisierte Maßnahmen der Qualifizierung zu entwickeln und mit der zeitlichen Organisation der Kurzarbeit abzustimmen. Zukünftig sollten in den Betrieben, bei Kleinbetrieben auch betriebsübergreifend in Verbünden, Lernpromotor:innen (Dobischat et al., 2013) oder Weiterbildungsmentor:innen (IG Metall, 2018) eingeführt werden. Deren Aufgabe bestünde darin, Qualifizierungsbedarfe zu eruieren, die betrieblichen Akteure zu motivieren und gezielte Maßnahmen anzustoßen. Weitere Unterstützung lässt das gerade vom Bundestag gestärkte Initiativrecht für Betriebsräte bei der Weiterbildung erwarten. Außerdem sollten, dem Beispiel der Niederlande folgend, Betriebe, die Kurzarbeit beantragen, erklären, dass sie Beschäftigte zu Weiterbildungsmaßnahmen anregen.

Dr. Toralf Pusch leitet das Referat Arbeitsmarktanalyse am Wirtschafts- und Sozialwissenschaftlichen Institut (WSI) der Hans-Böckler-Stiftung in Düsseldorf.

Dr. Hartmut Seifert war bis 2009 Leiter der Abteilung WSI der Hans-Böckler-Stiftung.


Literatur

Bellmann, L., P. Gleiser, C. Kagerl, T. Koch, C. König, T. Kruppe, J. Lang, U. Leber, L. Pohlan, D. Roth, M. Schierholz, J. Stegmaier und A. Aminian (2020), Weiterbildung in der Covid-19-Pandemie stellt viele Betriebe vor Schwierigkeiten, IAB Forum, https://www.iab-forum.de/weiterbildung-in-der-covid-19-pandemie-stellt-viele-betriebe-vorschwierigkeiten (13. Juli 2021).
Berger, K. (2015), Betriebsräte im Handlungsfeld betrieblicher Weiterbildung, in K. Berger, R. Jaich, B. Mohr, S. Kretschmer, D. Moraal und H. U. Nordhaus (Hrsg.), Sozialpartnerschaftliches Handeln in der betrieblichen Weiterbildung.
Bilger, F. und B. Käpplinger (2017), 19. Barrieren für die Bildungsbeteiligung Erwachsener, in Weiterbildungsverhalten in Deutschland 2016: Ergebnisse des Adult Education Survey (AES) (DIE Survey), 265–275, Deutsches Institut für Erwachsenenbildung.
Bundesgesetzblatt (2018), Gesetz zur Stärkung der Chancen für Qualifizierung und für mehr Schutz in der Arbeitslosenversicherung, Qualifizierungschancengesetz, http://www.bgbl.de/xaver/bgbl/start.xav?startbk=Bundesanzeiger_BGBl&jumpTo=bgbl118s2651.pdf (13. Juli 2021).
Christ, J. und S. Koscheck (2021), Auswirkungen der Corona-Pandemie auf Weiterbildungsanbieter. Vorläufige Ergebnisse der wbmonitor Umfrage 2020, https://www.bibb.de/de/12164.php (13. Juli 2021).
Crimmann, A. und F. Wießner (2009), Wirtschafts- und Finanzkrise: Verschnaufpause dank Kurzarbeit, IAB-Kurzbericht, 14/2009.
Deeke A Konjunkturelle Kurzarbeit — Was kann bei vorübergehendem Arbeitsausfall bewirkt werden? WSI-Mitteilungen 2009 62 8 446 452 10.5771/0342-300X-2009-8-446
Dobischat, R., K. Düsseldorf und H. Seifert (2013), Qualifizierung zum Lernpromotor: Ein erfolgreicher Ansatz zur Förderung der Weiterbildung in Klein- und Mittelbetrieben (KMU), https://www.mat-gmbh.de/files/content/mat/Publikationen/Qualifizierung%20zum%20Lernpormotor.pdf (13. Juli 2021).
Emmler, H. (2021), HBS-Erwerbspersonenbefragung, Welle IV: Fragebogen und Codebuch, WSI-Datenreport.
Herzog-Stein, A., P. Nüß, L. Peede und U. Stein (2021), Germany’s Labour Market in Coronavirus Distress — New Challenges to Safeguarding Employment, IMK Working Paper, 209.
IG Metall (2018), Gewerkschaftliche Weiterbildungsmentoren: Weiterbildung funktioniert nur mit Vertrauensleuten, https://wap.igmetall.de/17989.htm (13. Juli 2021).
Kruppe, T., J. Lang und U. Leber (2021), Nur jeder zehnte Betrieb nutzt die Weiterbildungsförderung der Bundesagentur für Arbeit, IAB Forum, https://www.iab-forum.de/nur-jeder-zehnte-betrieb-nutzt-die-weiter-bildungsfoerderung-der-bundesagentur-fuer-arbeit/ (13. Juli 2021).
Kruppe, T. und C. Osiander (2020), Kurzarbeit in der Corona-Krise: Wer ist wie stark betroffen?, IAB-Forum, https://www.iab-forum.de/kurzarbeit-in-der-corona-krise-wer-ist-wie-stark-betroffen/?pdf=16967 (13. Juli 2021).
Leifels, A. (2021), Weiterbildung bricht in der Krise ein — Bedarf an Digitalkompetenzen wächst, KfW Research, Fokus Volkswirtschaft, 329.
OECD (2021), Continuing Education and Training in Germany: Getting Skills Right, OECD Publishing.
Osiander C Stephan G Was beeinflusst die Weiterbildungsbereitschaft von Beschäftigten? Befunde aus einer Vignettenstudie Industrielle Beziehungen 2018 27 3 336 359
Pusch, T. und H. Seifert (2021), Kurzarbeit — mehr als eine Beschäftigungsbrücke, WSI Policy Brief, 53.
Schönfeld, G. und F. Behringer (2017), Betriebliche Weiterbildung, in Weiterbildungsverhalten in Deutschland 2016: Ergebnisse des Adult Education Survey (AES) (DIE Survey), 56–73, Deutsches Institut für Erwachsenenbildung.
Seyda, S. und B. Placke (2020), Erfahrung mit E-Learning erleichtert Weiterbildung während der Corona-Krise, IW-Kurzbericht, 117, https://www.econstor.eu/bitstream/10419/228813/1/IW-Kurzbe-richt-2020-117.pdf (13. Juli 2021).
Statistisches Bundesamt (2017), Berufliche Weiterbildung in Unternehmen. Fünfte Europäische Erhebung über die berufliche Weiterbildung in Unternehmen (CVTS5) 2015, https://www.destatis.de/DE/Themen/Gesellschaft-Umwelt/Bildung-Forschung-Kultur/Weiterbildung/Publikationen/Downloads-Weiterbildung/weiterbildung-unternehmen-5215201159004.pdf (13. Juli 2021).
